# Dermatology for the internist: optimal diagnosis and management of atopic dermatitis

**DOI:** 10.1080/07853890.2021.2004322

**Published:** 2021-11-17

**Authors:** Shanthi Narla, Jonathan I. Silverberg

**Affiliations:** a Department of Dermatology, St. Luke’s University Health Network, Easton, PA, USA; b Department of Dermatology, The George Washington University School of Medicine and Health Sciences, Washington, DC, USA

**Keywords:** Atopic dermatitis, internist, comorbidities, screening, mental health, depression, cardiovascular, food allergies, sleep, racial disparities, autoimmune conditions, diagnosis, treatment, environmental factors, bleach baths, infections

## Abstract

Internists are front-line health care providers that commonly provide the first encounter to patients for dermatological conditions, especially atopic dermatitis (AD). Internists need to be comfortable with managing mild-moderate AD in their practices. Criteria and guidelines established in dermatology literature are available to help the general practitioner diagnose and treat AD. AD is a systemic disease associated with multiple cutaneous and extra-cutaneous comorbidities that warrant screening by internists, especially mental health conditions. Environmental factors may play a role in the development or worsening of AD; however, there is currently no strong evidence to guide specific population- or clinic-based interventions for their avoidance. While food allergies are common in AD patients, the role of food allergens as an exacerbating factor for AD is controversial. Before starting any dietary modifications, careful evaluation should be performed by an allergist. If the patient is not well-controlled despite adequate topical therapies or is experiencing severe/worsening disease, early referral to dermatology is warranted to rule out confounding diagnoses and/or escalation to systemic therapies. Finally, it is important to recognise the racial disparities present in AD and address these when formulating treatment plans.

**Key messages:**
- Confounding dermatoses, either instead of or in addition to AD, should be considered in treatment-refractory AD, and the appropriate workup may be initiated while awaiting dermatology referral.
- AD patients have multiple cutaneous and extra-cutaneous comorbidities that warrant screening by internists, especially mental health conditions.

## Introduction

Atopic dermatitis (AD) is a heterogeneous chronic inflammatory skin disease associated with multiple cutaneous signs (e.g. redness, swelling, thickening, scaling), symptoms (e.g. itch, pain, sleep disturbance [SD]), and impacts on quality of life (QOL; e.g. impacts on activities of daily living, social interactions, and leisure activities). There are an estimated 8.4 million children [1] and 16.5 million adults in the U.S [2] who have AD, yet only 10,845 dermatologists [1,3–9]. As such, most patients with AD are managed by internists and are not able to see a dermatologist [10].

Most healthcare visits for AD in the United States occur with primary care providers (PCPs), with fewer visits to dermatologists, and much fewer to allergists. Moreover, the proportion of AD visits to PCPs appears to be increasing over time, with concomitantly fewer visits to dermatologists. Though, the nature of AD visits was different between PCPs (more acute visits) and dermatologists (more chronic visits). This may be explained by the long-standing relationship between PCPs and patients or the inability of patients to get in quickly enough to see a dermatologist for an acute flare [11]. Therefore, internists need to be able to evaluate, diagnose, and treat AD, and screen for associated comorbidities within the scope of their practices. Further, it is important to know when to refer patients to a dermatologist. This review will address key issues for internists regarding the diagnosis and management of AD.

## Pathophysiology

### Genetics

AD is caused by a convergence of genetic, immunologic, skin-barrier, microbiome, environmental, and behavioural factors [12]. Parental history of AD is associated with a 2–3 fold increased risk of having children with AD [13]. Monozygotic twins have a 3-fold higher concordance rate than dizygotic twins [14]. The strongest genetic risk factor for AD is loss-of-function mutations of the filaggrin (FLG) gene [15]. FLG null mutations lead to a deficiency of natural moisturising factors (amino acids that play an integral role in skin barrier function) [16], xerosis (dryness) in AD [17], and epidermal barrier dysfunction [18]. It is noteworthy that most AD patients do not have FLG mutations, and 4 in 10 people with FLG null mutations do not have AD [19].

### Immunology

In the acute and chronic phases of AD, T-helper (Th)2 effector cells produce interleukins (IL)-4, 5, 13, and 31 and Th22 cells producing IL-22 and S100A proteins predominate, with increased IL-17 production in some patients [20,21]. IL-31 is implicated in itch and skin-homing of T cells [20,22]. In chronic AD, there is also increased IL-12 production, Th1 cells, and related cytokines (e.g. interferon-gamma) [21,22].

### Skin-barrier

Keratinocytes (epithelial cells of the skin) differentiated in the presence of IL-4 and IL-13 exhibit reduced FLG gene expression, even in patients without FLG null mutations, and also downregulate loricrin and involucrin. Loricrin, involucrin, and filaggrin are important proteins in the cornified cell envelope, which is integral in skin-barrier formation [23].

### Microbiome

*Staphylococcus aureus* (*S. aureus)* colonises *the* skin of 30–100% of AD patients, but only 20% of healthy people [24]. Further, analysis of inflamed skin from AD patients has shown a reduction of microbial diversity during an AD flare, with decreases in *Streptococcus*, *Corynebacterium*, and *Cutibacterium* and the phylum Proteobacteria towards *S. aureus* [25].

It is unclear if the microbial change is a cause or effect of barrier-dysfunction and cutaneous inflammation in AD. *S. aureus* colonisation may precede flares and facilitate inflammation in AD [26]. In contrast, IL-4 and IL-13 inhibit the production of cutaneous antimicrobial peptides, predisposing AD skin to Staphylococcus infections, and Th2 cells facilitate the binding and colonisation of *S. aureus* [24].

### Environmental factors

Multiple environmental factors play a role in AD. However, identifying definitive factors proves challenging given disease heterogeneity, lack of randomised controlled trials, conflicting evidence, and complex gene-environment interactions.

Some environmental exposures found to be associated with increased AD include in-utero exposure to smoking, alcohol, antibiotics, gestational diabetes, and maternal stress while in-utero [27]. Whereas, maternal consumption of fish oil, omega-3 fatty acids, and probiotics may be protective [27]. Increased risk of AD from in-utero or prenatal exposure to antibiotics may be related to the hygiene hypothesis, i.e. decreased exposure to certain microbes in early life may steer the immature immune system towards an increased incidence of autoimmune and allergic diseases [28]. Early-life exposures potentially associated with increased AD include low levels of vitamin C and D in breast milk, increased birth weight, and caesarean section births [27].

It remains unclear whether exclusive breastfeeding reduces AD risk [29–31]. A recent systematic review (SR) and meta-analysis of 45 studies from 20 countries showed no associations between early introduction of cow’s milk or cow’s milk formula and development of AD; however, the SR was comprised of fairly low-quality evidence [32]. One controlled trial provided weak evidence that early introduction of allergenic foods around 4 months of age may reduce AD risk [33].

Other studies have suggested that diet may play a role in AD, especially in children and adolescents [34]. AD may be increased in children who consume significant amounts of fast food or a diet rich in fats. A survey of 169 patients found that elimination of white flour products, gluten, and nightshades, and the addition of vegetables, organic foods, and fish oil led to improvement of their AD symptoms [35].

Other factors associated with increased incidence of AD in children or adults include decreased outdoor temperature, low humidity, low ultraviolet B radiation index, increased indoor heating, high levels of air pollution, common use of household products, dust mites, and hard water use, while early life exposures to pet dander may be protective [27]. While these environmental factors may play a role in the development or worsening of AD, there is currently no strong evidence to guide specific population- or clinic-based interventions for their avoidance [27].

## Diagnosis

### Morphology

AD is a heterogeneous and polymorphic disease. Acute lesions can be characterised by erythema (redness), exudation (oozing), papules (raised bumps), scales, and crusts. In comparison, chronic lesions may exhibit infiltrated erythema, lichenification (thickened, leathery skin with accentuation of skin lines), scales, crust, and prurigo (firm, dome-shaped bumps secondary to picking of intense itch) [36].

An SR and meta-analysis (*n* = 101 studies) were performed to determine the prevalence of AD characteristics and differences by region and age. The most prevalent AD features were pruritus (itch) and xerosis (dryness). Other common features included lichenification, a course influenced by emotional and/or environmental factors, flexural involvement, early onset of disease, and worsening of itch with sweating [37].

Regional differences in AD characteristics were identified. Xerosis was amongst the top 5 most commonly reported features in all regions, except Southeast Asia (SEA). This may be because SEA has a more humid tropical climate. Flexural involvement was common in Australia, Africa, SEA, East Asia (EA), India, and Europe, but less common in the Americas and Iran. Extensor involvement was more common in India, followed by EA and Iran. Head, face, and neck involvement was most common in Iran, Africa, and the Americas, followed by Australia, EA, Europe, and India, and least common in SEA. Dennie-Morgan fold (extra line in the skin below the lower eyelid) was most common in India, followed by Europe and Africa, and less common in Iran, the Americas, SEA, and EA. Orbital darkening, papular lichenoid lesions (shiny, flesh-coloured or reddish-brown, flat-topped bumps), and palmar hyperlinearity (increased number and accentuation of lines on palms) were more commonly reported in Africa [37].

Age-related differences in AD presentation were observed. Compared to adult studies, paediatric studies reported a higher prevalence of dermatitis affecting eyelid, auricle, and ventral wrist; exudative eczema; seborrhoeic dermatitis-like features. Adults had a higher prevalence of lichenification, erythroderma, disease course influenced by emotions and/or environmental factors, ichthyosis (widespread and persistent thick, dry, “fish-scale” skin), palmar hyperlinearity, keratosis pilaris, hand and foot dermatitis, dyshidrosis, prurigo nodularis, and papular lichenoid lesions [37]. Keratosis pilaris is a common skin condition that causes dry, rough patches and tiny bumps, often on the upper arms, thighs, cheeks, or buttocks.

Morphology and distribution of eczema in AD varies with age: infants (birth to 2 years of age) commonly present with red papules on cheeks, forehead, and scalp. Patients of all ages commonly exhibit dry skin, lichenified papules, and plaques in flexural areas (creases of elbows, knees, wrists, ankles, and anterior neck). Adults commonly present with eczema on the face and neck, upper arms and back, hands, feet, fingers, and toes [21].

### Diagnostic criteria

AD is diagnosed clinically based on history, symptoms, lesional morphology, and distribution [12]. Recent studies show that AD is common in children and adults, with paediatric disease commonly persisting into adulthood [38]. Allergic rhinitis and hand eczema [39], already persistent disease, and/or more severe disease [40] were associated with increased persistence of paediatric AD into adulthood.

In 1980, Hanifin and Rajka developed the earliest and most recognised criteria for diagnosing AD which requires 3 of 4 major and 3 of 23 minor criteria to be met [41]. The United Kingdom (UK) Working Party criteria requires itchy skin plus 3 of 5 criteria to be met, and was developed from the Hanifin-Rajka criteria to serve as a tool for non-dermatologists to diagnose AD. In 2003, the American Academy of Dermatology (AAD) further streamlined the Hanifin-Rajka criteria for clinical practice and all ages [12]. AAD criteria require pruritus, eczema with typical morphology or age-specific patterns, and chronic or relapsing history. Typical patterns include facial, neck, and extensor involvement in infants and toddler, flexural dermatitis at any age, and sparing of groin and axillae. Important features that support AD diagnosis include early age of onset, personal or family history of atopy, immunoglobulin E reactivity, and xerosis; though, none of these are essential [12]. Hanifin-Rajka and UK Working Party criteria were previously validated across multiple populations [42–50], while AAD criteria were not.

Most importantly, all criteria highlight the importance of excluding other conditions such as scabies, seborrhoeic dermatitis, irritant or allergic contact dermatitis (ACD), ichthyosis, cutaneous T-cell lymphoma (CTCL), psoriasis, photosensitivity dermatoses, immunodeficiencies, and erythroderma of other causes [12,51]. Immunodeficiencies include Netherton syndrome, severe combined immunodeficiency, Wiskott-Aldrich syndrome, Autosomal dominant hyper-IgE syndrome (AD-HIES), Autosomal recessive HIES, Immune dysregulation polyendocrinopathy, enteropathy X-linked syndrome (IPEX), and acrodermatitis enteropathica [51]. Acrodermatitis enteropathica should be considered in infants with periorificial and acral dermatitis accompanied by alopecia, diarrahea, and failure-to-thrive. Workup for acrodermatitis enteropathica includes measuring plasma zinc concentration levels and screening for the SLC39A4 gene mutation [52–54].

The pathognomonic sign of scabies is the burrow- a short, wavy, scaly, grey line on the skin surface. Burrows are often found on hands and feet, particularly in finger web spaces, thenar and hypothenar eminences, and wrists. Scabies is generally characterised by intractable pruritus that is worse at night with lesions affecting finger web spaces, thenar and hypothenar eminences, wrists, buttocks, axillae, abdomen (around the umbilicus), and genitals. Scabies should be considered in any adult with widespread eczema or pruritus of new-onset. In infants and young children, scabies often affects the face, head, neck, scalp, palms, and soles, and there is often generalised skin involvement. In infants, the most common lesions are papules and vesicopustules present on palms and soles. Itching in several family members over the same time period also supports the diagnosis of scabies. Definitive diagnosis relies on microscopic identification of mites or eggs from skin scrapings of a burrow. Even if the diagnosis of scabies cannot be microscopically confirmed, treatment should be empirically initiated if the clinical suspicion is high [55].

Seborrhoeic dermatitis (SD) generally appears on the scalp and face and much less commonly on the chest, back, axilla, and groin. SD can be associated with human immunodeficiency virus infection and neurologic disease, e.g. stroke, Parkinson’s disease. SD commonly presents in infants with thick white or yellow greasy scales on the scalp. In adolescents and adults, SD typically presents as flaky, greasy, red plaques on the scalp, nasolabial folds, ears, and eyebrows. SD is diagnosed clinically based on the lesion location and appearance. First-line SD treatment is topical antifungal agents [56].

Clinical presentations of contact dermatitis vary by the causative agent and affected areas of the skin. Irritant dermatitis is characterised by pruritus, burning, and pain; ACD is characterised predominantly by pruritus [57]. A meta-analysis including 28 studies found that ≥20% of the general population has contact hypersensitivity to common allergens, e.g. nickel, fragrances, and cobalt [58]. Contact dermatitis usually manifests as redness and scaling with relatively well-demarcated borders. In the acute stage, clinical findings include redness, papules, swelling, and blisters. In the subacute and chronic stages, lichenification, fissuring, and scaling can be seen. In most cases, lesions are localised to the site of contact; patchy or diffuse lesions can also occur [58]. Additionally, there are some variants of contact dermatitis with atypical clinical features, such as targeted erythema, purplish papules, and plaques and hyperpigmentation. Hands, face, neck, and perianal or genital areas are commonly involved, although any area can be affected [59–63]. In contrast to contact dermatitis, AD is more widespread and much more commonly affects flexural areas. Diagnosis of contact dermatitis is confirmed by observing whether dermatitis resolves with avoidance of suspected allergens. If the allergen is unknown or empiric avoidance and topical treatment do not resolve dermatitis, patch testing may be indicated [57].

CTCL often presents with red scaly patches or plaques that can mimic AD or psoriasis. Lesions are generally confined to non-sun-exposed areas, e.g. buttocks, medial thighs, and breasts. CTCL often presents with dermatitis that is follicle-centric or atrophic plaques with a cigarette paper-like consistency [64]. A thorough history including progression of systemic symptoms should be obtained, including fevers, chills, malaise, fatigue, and weight loss. When CTCL is suspected, lymph nodes examination should be performed [64] with a low threshold to biopsy. Multiple biopsies and repeat biopsy may be needed for accurate diagnosis. Laboratory testing should include a complete blood count with differential; flow cytometry (leukemia/lymphoma blood panel) and T-cell receptor rearrangements studies of skin and blood may aid in diagnosis [64].

Other disorders in the differential diagnosis of refractory AD include non-bullous/urticarial-phase pemphigoid (NBP), dermatomyositis, dermatitis herpetiformis, and cutaneous lupus erythematosus [65]. NBP is an under-recognised variant of pemphigoid with long diagnostic delays. In NBP, patients present with pruritus and a wide spectrum of skin manifestations that resemble other inflammatory skin diseases; notably there are often no blisters or urticarial plaques [66]. Diagnosis of NBP is based on skin biopsy sent for direct immunofluorescence microscopy or by circulating serum antibodies by indirect immunofluorescence microscopy (Bullous Pemphigoid 180 and 230) [66]. Dermatitis herpetiformis presents with diffuse, symmetrical, grouped polymorphic lesions consisting of red, urticarial plaques, papules, herpetiform vesicles, and blisters followed by erosions, excoriations, and hyperpigmentation [67]. Commonly involved sites include the extensor surfaces of the elbows (90%), knees (30%), shoulders, buttocks, sacral region, and face [67]. Lesional biopsy for haematoxylin and eosin staining and perilesional skin biopsy for direct immunofluorescence along with specific serologic studies (e.g. IgA endomysial antibody, IgA tissue transglutaminase antibody) can be performed [67]. Referral to a dermatologist may be warranted to rule out all of the conditions discussed above in the differential for refractory AD.

## Treatment guidelines

Multiple guidelines recommend a step-up approach to manage AD based on disease severity, and whether a patient is undergoing an acute flare [68]. For mild AD, acute flares can be managed with low-medium potency topical corticosteroids (TCS), calcineurin inhibitors (TCI), or a phosphodiesterase E4 (PDE4) inhibitor twice daily for 3–7 days beyond clearance of acute lesions. Maintenance therapy includes moisturising and trigger avoidance (e.g. proven allergens and common irritants such as soaps, wool, and temperature extremes). Other common triggers of itch in AD include heat, sweat, tight clothing, fragrances, boredom, talking about itch, stress, weather change, sunlight, humid air, and dry air [69].

For moderate AD, acute flares can be managed with medium-high potency TCS twice daily for 3–7 days beyond clearance. Maintenance therapy can also include the application of low potency TCS 1–2× daily (including face) or a medium potency TCS 1–2× weekly (except face) to areas prone to flaring [68]. TCI or PDE4 inhibitors can be applied 1–2× daily or 2–3× weekly. For moderate to severe disease, if no improvement is noted in 7 days, factors that should be considered include non-adherence, misdiagnosis, contact allergy to medications, or referral to a dermatologist [68].

For severe AD, additional considerations include referral to an AD specialist (dermatologist or allergist) for treatment with advanced therapies, e.g. biologics (dupilumab), systemic immunosuppressants (cyclosporine, methotrexate, corticosteroids), or phototherapy. Additionally, wet wraps or short-term hospitalisation should be considered to achieve control of severe flares that are not well controlled in the outpatient setting.

### Food allergies

Patients and caregivers are commonly concerned about foods being potential causes or triggers of AD. Across studies, the prevalence of food allergy in children with AD varies significantly between 20 and 80 percent due to different study populations, AD severities, and a lack of consistency of defining criteria for food allergy [70]. Up to 53% of children with AD have demonstrable positive food-specific IgE and/or positive skin prick tests (SPTs) with up to 15% demonstrating signs of food allergy on oral food challenge [71]. However, many of these positive tests are false positives without clinical relevance. Food challenge proven immunoglobulin E (IgE)-mediated food allergy (a more refined definition) may be present in up to one-third of moderate-severe AD patients [72]. In infants, cow’s milk, hen’s eggs, peanut, and soy and in older children, wheat, fish, tree nuts, and shellfish are the most common food allergens [73]. Birch-associated foods were also described as potential triggers of AD in children as well as in adults [73].

While food allergies are common in AD patients, the role of food allergens as exacerbating or triggering factors in AD is controversial. In 1978, Atherton *et al.* showed for the first time in a 12-week, double-blind, controlled crossover trial that elimination of cow’s milk and hen’s eggs lead to improvement in eczema in 14 of 20 children with AD [74]. According to guidelines established by the International Collaboration in Asthma, Allergy and Immunology, children with a history of an immediate reaction to a single food, or refractory moderate-to-severe AD with food being investigated as a potential trigger should be considered for a food allergy work-up. This highlights the potential role of food allergens as eczema triggers in moderate-severe AD and increased food allergy risk in AD patients [75]. Elimination should be guided by appropriate testing, establishing clinical relevance, and should result in significant clearing. Before starting dietary modifications, careful evaluation should be performed by an allergist to confirm causality between foods and AD exacerbations, as unnecessary dietary modifications may lead to nutritional deficiencies [76].

### Moisturisers and topical medications

Maintaining skin hydration and preventing transepidermal water loss are essential components of AD treatment [77]. A recent SR and meta-analysis demonstrated that daily bathing/showering was not associated with more severe AD than less frequent bathing/showering. In sensitivity analyses, daily bathing was associated with similar or greater improvements of Eczema Area and Severity Index (EASI) and SCORing Atopic Dermatitis (SCORAD) scores compared to less frequent bathing. Thus, regular bathing or showering should not be discouraged in AD patients [78]. For moderate-severe AD, patients should be instructed to bathe once daily, preferably before bedtime.

Warm (not hot) water for 10–15 min should be used. A gentle cleansing bar or wash (e.g. Dove^®^ Sensitive Skin Unscented Beauty Bar, Aquaphor^®^ Gentle Wash, AVEENO^®^ Advanced Care Wash, CeraVe™ Hydrating Cleanser, and Cetaphil^®^ Gentle Cleansing Bar) should only be used as needed. After finishing showering/bathing, the skin should be pat dry (do not rub) and immediately afterwards (within 1–2 min) moisturiser or topical medication should be applied. Fragrance-free moisturisers available in one-pound jars include Vaseline^®^ or Aquaphor^®^ ointment, Eucerin^®^ cream, Vanicream^®^, CeraVe^®^ cream or Cetaphil^®^ cream. In a study examining the use of moisturisers for the prevention of AD in high-risk newborns, petrolatum was found to be the most cost-effective moisturiser ($353/quality-adjusted life year) in the cohort [79]. When the clinical and cost-efficacy of a glycyrrhetinic acid-containing barrier repair cream (BRC-Gly, Atopiclair^®^), a ceramide-dominant barrier repair cream (BRC-Cer, EpiCeram^®^) and an over the counter petroleum-based skin protectant moisturiser (OTC-Pet, Aquaphor Healing Ointment^®^) were compared as monotherapy for mild-to-moderate AD in children, the results showed that there was no statistically significant difference in efficacy between the three groups at each time point studies, and the OTC-petroleum was at least 47 times more cost-effective [80,81]. Moisturisers should be used liberally at least three times daily. Moisturisers and sealers should not be applied over topical prescription medications as they will dilute the efficacy [81,82].

### Bleach baths

Bleach baths have been proposed as a potential adjunctive treatment in patients with moderate-severe AD [68]. Bleach baths are performed by filling a tub with warm water (∼40 gallons) and adding ½ cup of common bleach solution to the bathwater (or ¼ cup in half a tub of water). Any sodium hypochlorite 6% solution can be used (e.g. Clorox liquid bleach). Patients can soak in the chlorinated water for 10 min, rinse with fresh lukewarm water, followed by the immediate application of topical emollients or medications [83].

However, evidence for the efficacy of bleach baths is weak and suggests that bleach baths are no more effective than plain water baths. Huang *et al.* performed a randomised placebo-controlled study of 31 patients (ages 0.5–17 years) with moderate-severe AD and clinical signs of secondary bacterial infections. All patients received cephalexin for 14 days and were assigned to either receive intranasal mupirocin ointment and bleach baths or intranasal petrolatum ointment treatment and plain water baths for 3 months. Patients in the group that received dilute bleach baths and intranasal mupirocin treatment showed significantly greater reductions in EASI than in the placebo group at 1-month and 3-month visits [84].

However, subsequent randomised clinical trials (RCTs) demonstrated differing results. Recently, a systematic review and meta-analysis on the efficacy of bleach baths in reducing the severity of AD found that both bleach baths and water baths alone significantly reduced AD severity. Moreover, water baths were just as effective as bleach baths at 4 weeks in pooled analyses. There were no differences in *S. aureus* density in the patients that received bleach baths versus water. Therefore, the results suggested that the efficacy of bleach baths at reducing AD severity may be just from soaking in the water per se [85]. Further, at the recommended concentration of bleach (sodium hypochlorite 0.005%), *in vitro* studies showed no bactericidal effect. A concentration of at least 0.03% was needed to have a bactericidal effect against certain strains of *S. aureus*; however, this concentration is cytotoxic to human cells and is much greater than should be used clinically [86].

### Infections

Adults and children with AD have an increased likelihood of cutaneous infections, including erysipelas, cellulitis, herpes simplex, herpes zoster, warts, molluscum contagiosum, and eczema herpeticum [87–90]. AD patients may also have increased odds of extracutaneous, multiorgan, and systemic infections, including necrotising fasciitis, acute sinusitis, acute pharyngitis, bronchitis, croup, acute epiglottitis, pneumonia, aspergillosis infections, tuberculosis, meningitis, encephalitis, endocarditis, infectious arthropathies, enterocolitis, *clostridium* difficile, pyelonephritis, urinary tract infections, MSSA, MRSA, streptococcal, pseudomonas, mycobacterial, fungal, viral, influenza, and septicaemia [87,89,91–94]. Consequently, it is important for internists to maintain a high suspicion for various cutaneous and extracutaneous infections when treating AD patients.

## Screening for comorbidities

AD patients were also found to have increased hypertension [95], BMI and/or obesity [96,97], poor cardiovascular outcomes [98], type II diabetes mellitus [98], osteoporosis, and fracture of a bone or joint disease [99,100]. In a study examining cardiovascular risk in 387,439 U.K. adults with AD, during an average of five years of follow-up after initial diagnosis of AD, the risk of CV death was 30% higher in patients with severe eczema, heart failure was 70% higher, and unstable angina and atrial fibrillation were about 40% higher than in controls. Severe eczema was associated with a 20% increased risk of stroke. A dose-response effect was observed, with CV risks increasing from mild to moderate to severe eczema [101,102]. One study using modelling techniques found that a genetically determined increase in adiposity was associated with an increased risk of AD. In contrast, genetically determined increased risk of AD was not associated with a higher BMI. Consequently, these results suggest a possible causal role of adiposity in AD [103].

A population-based adult cohort study found that those with eczema had an increased risk of hip, pelvic, spinal, and wrist fractures. Fracture risk increased with increasing AD severity, with the strongest associations in people with severe eczema for spinal, pelvic, and hip fractures. Most importantly, all of these associations remained after adjusting for oral glucocorticoid treatments [104]. In a study of 125 adult patients with moderate-severe AD, osteoporosis was documented in 6 (4.8%) patients and osteopenia (32.8%) in 41 patients. 30.4% of the patients had low bone mineral density, with more men (43.8%) than women (16.4%) affected. These results were independent of the cumulative dose of topical and/or oral corticosteroids used within 5 years of the study [105]. Internists need to maintain a high suspicion for the development of these comorbid conditions in their patients and ask for a detailed review of systems at each visit.

### Skin pain

Skin pain is a lesser-known sensory symptom of the condition and one that is less recognised by dermatologists and treatment guidelines [106]. Previous studies have shown that patients with AD use different words to describe their experience of skin pain such as “burning,” “stinging,” “tingling,” and “pinprick-like” [106–109]. Recent evidence demonstrates that skin pain is not adequately assessed or managed by current standards of AD therapy. However, skin pain severity should be considered relevant when evaluating treatment efficacy and is therefore important to assess in clinical practice [110].

### Mental health

AD is associated with a considerable psychological burden in patients and caregivers. Previous studies showed that mothers of children with AD had higher stress scores than mothers of children without AD and children with other chronic disorders, e.g. insulin-dependent diabetes and significant deafness [111].

AD is associated with increased depression in children [112] and adults, anxiety in adults [113], and suicidal ideation in adolescents [114] and adults [115]. Several studies showed associations between AD and completed suicide [116]. AD was found to be associated with higher odds of the primary admission for multiple mental health (MH) disorders, including mood disorders, schizophrenia, and developmental disorders [117]. Pruritus, psychological stress, social isolation, depression, anxiety, and other mental health disorders can result in a vicious cycle in AD patients [116]. AD is also associated with attention-deficit (hyperactivity) disorder, conduct disorder, and autism [112,118,119].

Despite the high burden of depression in adults and children with AD, screening for depressive symptoms in the outpatient setting is very low. Analysis of the 2006–2015 National Ambulatory Medical Care Survey found that only 1.6% of AD patient encounters included screening for depression, with similar rates in dermatologists and non-dermatologists and all AD severities [120]. Healthcare providers should consider screening AD patients for MH comorbidities. Patient Health Questionnaire-9 and Hospital Anxiety and Depression Scale are validated in AD and are feasible for use in clinical practice to screen for depression and anxiety [121–123].

### Sleep

Sleep disturbances occur in 47–80% of children and 33–87.1% of adults with AD and increase with AD severity [124–126]. Commonly reported sleep disturbances include difficulty falling asleep, frequent night-time awakenings, and excessive daytime sleepiness [126]. Reducing sleep disturbances is a key treatment target in AD management. The most commonly used sleep aids in AD are first-generation anti-histamines. These can cross the blood-brain barrier resulting in a sedating effect, although current evidence shows they have no efficacy at improving itch or skin lesions in AD [127]. Tolerance to anti-histamines commonly occurs after 4–7 days of treatment. Anti-histamines have many off-target effects at higher doses. Anticholinergic side-effects, e.g. blurred vision and dry mouth, can occur. Sedating antihistamines can ultimately *worsen* sleep quality, decrease rapid eye movement (REM) sleep, and impair daytime cognitive function and work efficiency. Thus, first-generation antihistamines should not be used to treat itch or dermatitis, and only be used for short-term management of sleep disturbances in AD [126].

In a double-blind placebo-controlled RCT, oral melatonin 3 mg daily for 4 weeks resulted in marked reductions of sleep-onset latency and modest improvement of childhood AD severity; no adverse events were reported [128]. Melatonin has a good safety profile and may be a favourable choice in both adults and children to help with sleep. Other general sleep hygiene measures, such as developing positive and consistent bedtime routines, should be recommended [129]. Other sleep-promoting agents, e.g. benzodiazepines, chloral hydrate, and clonidine, lack supporting evidence [126] and are not recommended in AD.

## AD in diverse populations

There are multiple differences in the clinical presentation of AD across diverse patient populations. For example, AD is classically described as pruritic, erythematous plaques with fine overlying scale affecting flexural surfaces (e.g. antecubital and popliteal fossae). This presentation tends to be more common in white patients. In contrast, Asian patients tend to have more well-demarcated lesions (e.g. nummular or psoriasiform) and more scaling and lichenification [130,131]. African American patients present with more extensor involvement [132]. Perifollicular accentuation and scattered distinct papules on the extensors and trunk, and lichen-planus like presentation are much more common in darker-skinned individuals [133]. Erythema in darker-skinned individuals may appear more purple or brown and is commonly underappreciated. Black patients have increased lichenification and prurigo nodules possibly due to more severe pruritus and chronic scratching [134].

Multiple racial disparities exist for AD in the United States. Black children have a higher prevalence of AD [135], have increased AD severity [1], more emergency department visits [136] and inpatient hospitalizations [137], and were less likely to receive standard-of-care topical and systemic therapies for AD [138]. Genome-wide association studies suggested that gene-environment interactions only partially explain observed differences in AD severity between racial and ethnic groups, but not AD susceptibility overall [139]. Racial disparities in AD may be related to poor access to healthcare and lack of awareness by internists and other healthcare providers about the heterogeneous clinical presentations of AD in diverse patient populations.

## Conclusion

AD is a highly prevalent and burdensome disease in adults and children worldwide. Most AD patients have mild disease and should be managed in the primary care setting. AD patients have multiple cutaneous and extra-cutaneous comorbidities that warrant screening by internists, especially mental health conditions. It is important for internists to be comfortable managing mild-moderate AD in their practices and referring moderate-severe AD to specialists for advanced therapy.

## Data Availability

Data sharing is not applicable to this article as no new data were created or analysed in this study.

## Abbreviations

AD: atopic dermatitis
TCS: topical corticosteroids
TCI: topical calcineurin inhibitors
US: United States
AAD: American Academy of Dermatology
FLG: filaggrin
IL: interleukin
RCT: randomised clinical trial
MH: mental health
HRQOL: health-related quality of life
DLQI: Median Dermatology Life Quality Index
AA: African American
SEA: Southeast Asia
EA: East Asia
SD: sleep disturbances
QOL: quality of life
PCP: primary care physician
SR: systematic review
PDE4: phosphodiesterase E4
OTC: over the counter
CTCL: cutaneous T-cell lymphoma
ACD: allergic contact dermatitis
ACD: allergic contact dermatitis
EASI: Eczema Area and Severity Index
SCORAD: SCORing Atopic Dermatitis
MH: mental health
ACD: allergic contact dermatitis
SD: seborrhoeic dermatitis
